# Supplementary material for: Membrane-cytoskeletal crosstalk mediated by myosin-I regulates adhesion turnover during phagocytosis
Source: Nat Commun. 2019 Mar 19;10:1249. doi: 10.1038/s41467-019-09104-1 (PMC6425032; doi:10.1038/s41467-019-09104-1)
Supplement: Supplementary file 1 — Supplementary Information [file 41467_2019_9104_MOESM1_ESM.pdf]

Supplementary data - Barger et al.

Membrane-cytoskeletal crosstalk mediated by myosin-I regulates adhesion turnover during phagocytosis

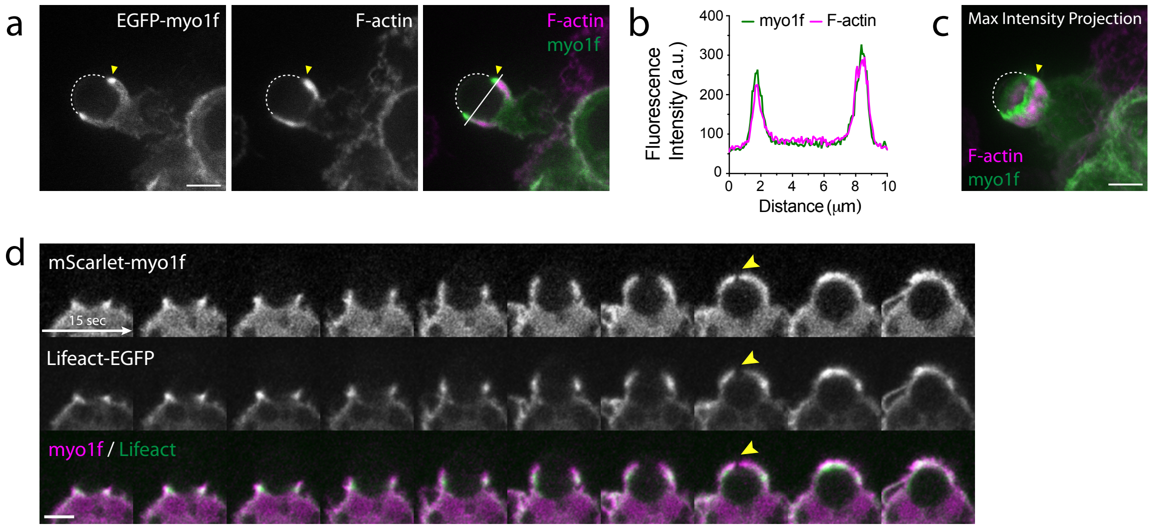

Supplementary Fig. 1: Myo1f localizes to the phagocytic cup.
a) Representative confocal section of a RAW264.7 macrophage transfected with EGFP-myo1f engulfing a 6 μm IgG-coated bead and stained with fluorescently-labeled phalloidin. Yellow arrowhead points to the phagocytic cup, and the bead is outlined by a dotted white line. Scale bar, 5 μm.
b) Line scan of EGFP-myo1f and F-actin intensity along the white line in (a) Merge to illustrate actin and myo1e colocalization at the phagocytic cup.
c) Maximum intensity projection of (a) Merge shows that myo1f precedes actin at the leading edge of the phagocytic cup.
d) Time-lapse montage of RAW macrophage expressing mScarlet-myo1f and Lifeact-EGFP engulfing 8 μm IgG-coated bead. Yellow arrowhead points to myo1f preceding F-actin, particularly at cup closure. Scale bar, 5 μm. See Supplementary Movie 2.

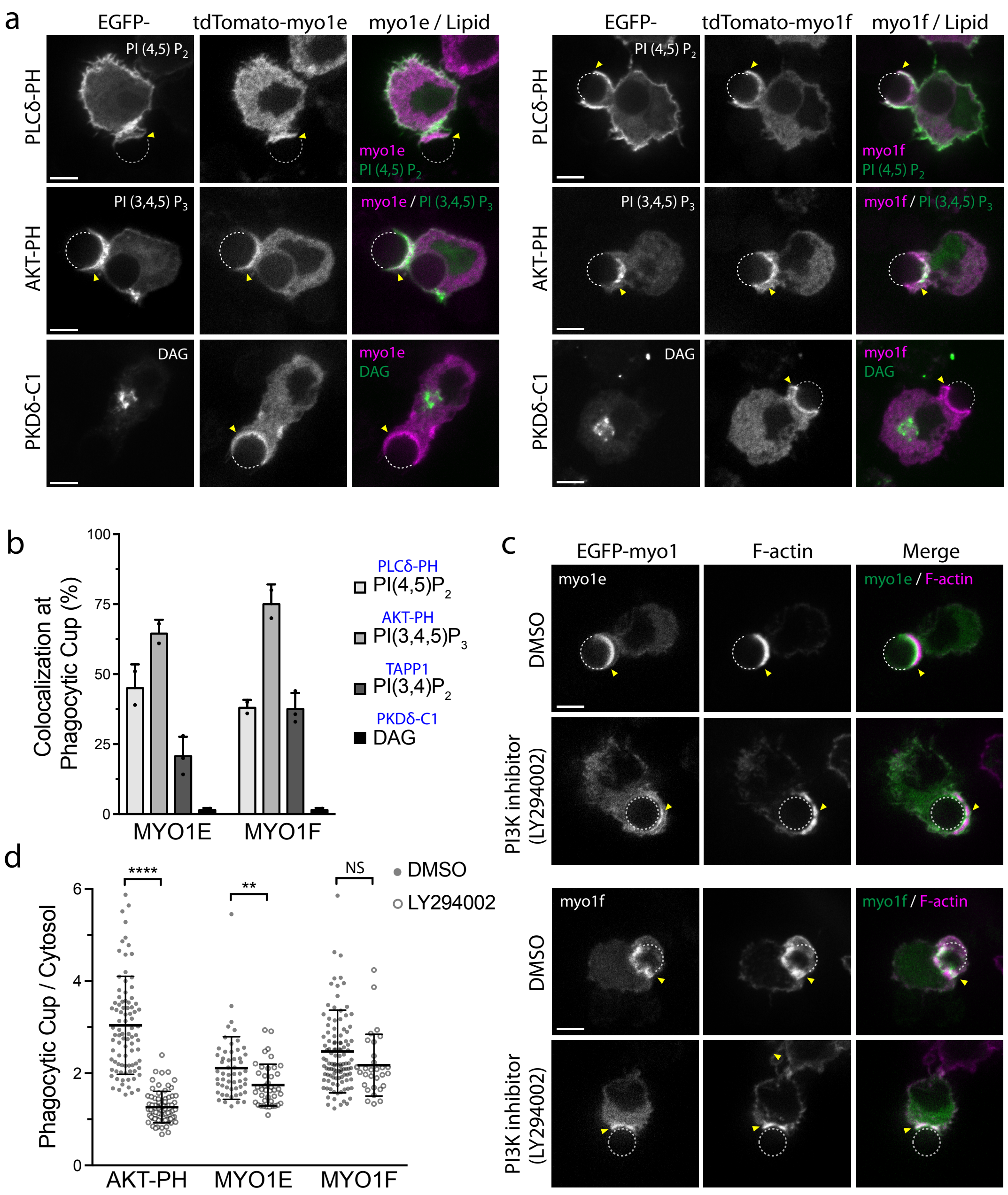

Supplementary Fig. 2: The relationship between myo1e and myo1f localization at the phagocytic cup and phosphoinositides.

a) Representative confocal sections of phagocytic cup localization of tdTomato-myo1e/f and EGFP-tagged lipid sensors in RAW macrophages. Yellow arrowheads point to the phagocytic cup, and the bead is outlined by a dotted white line. Scale bar, 5 µm.

b) Percentage of cells (mean ± SD) in which myo1e/myo1f colocalized with specific phospholipid markers at the phagocytic cup. EGFP-PKDδ-C1 (DAG sensor) was used as a negative control. Data from 2-3 independent experiments (>45 cups analyzed per lipid sensor per myosin construct).

c) Myo1e/f localize less robustly to the phagocytic cup when PI3K is inhibited. RAW macrophages transfected with EGFP-myo1e/f were pretreated with DMSO or 50 µM PI3K inhibitor LY294002 for 30 minutes, then challenged to engulf 6 µm IgG-coated beads. Cells were then fixed and stained with phalloidin to label F-actin. Images are representative confocal sections. Yellow arrowheads point to the phagocytic cup, and the bead is outlined by a dotted white line. Scale bar, 5 µm.
d) Ratio of myo1e/f enrichment at the phagocytic cup compared to the cytosol (mean ± SD) in transfected RAW macrophages treated with DMSO or LY294002. AKT-PH construct enrichment, shown on the same graph, was used as a control for PI3K inhibition. Peak fluorescence intensity levels at the cup and average fluorescence intensity in the cytosol were determined by line scan of single confocal slice. Data pooled from 2 independent experiments. AKT-PH: $p=<0.0001$, unpaired t-test (N = 90 DMSO cups, 72 LY294002 cups); MYO1E: $p=0.0035$, unpaired t-test (N = 54 DMSO cups, 41 LY294002 cups); MYO1F: $p=0.0935$, unpaired t-test (N = 111 DMSO cups, 30 LY294002 cups), **=$p<0.05$, ****=$p<0.0001$.

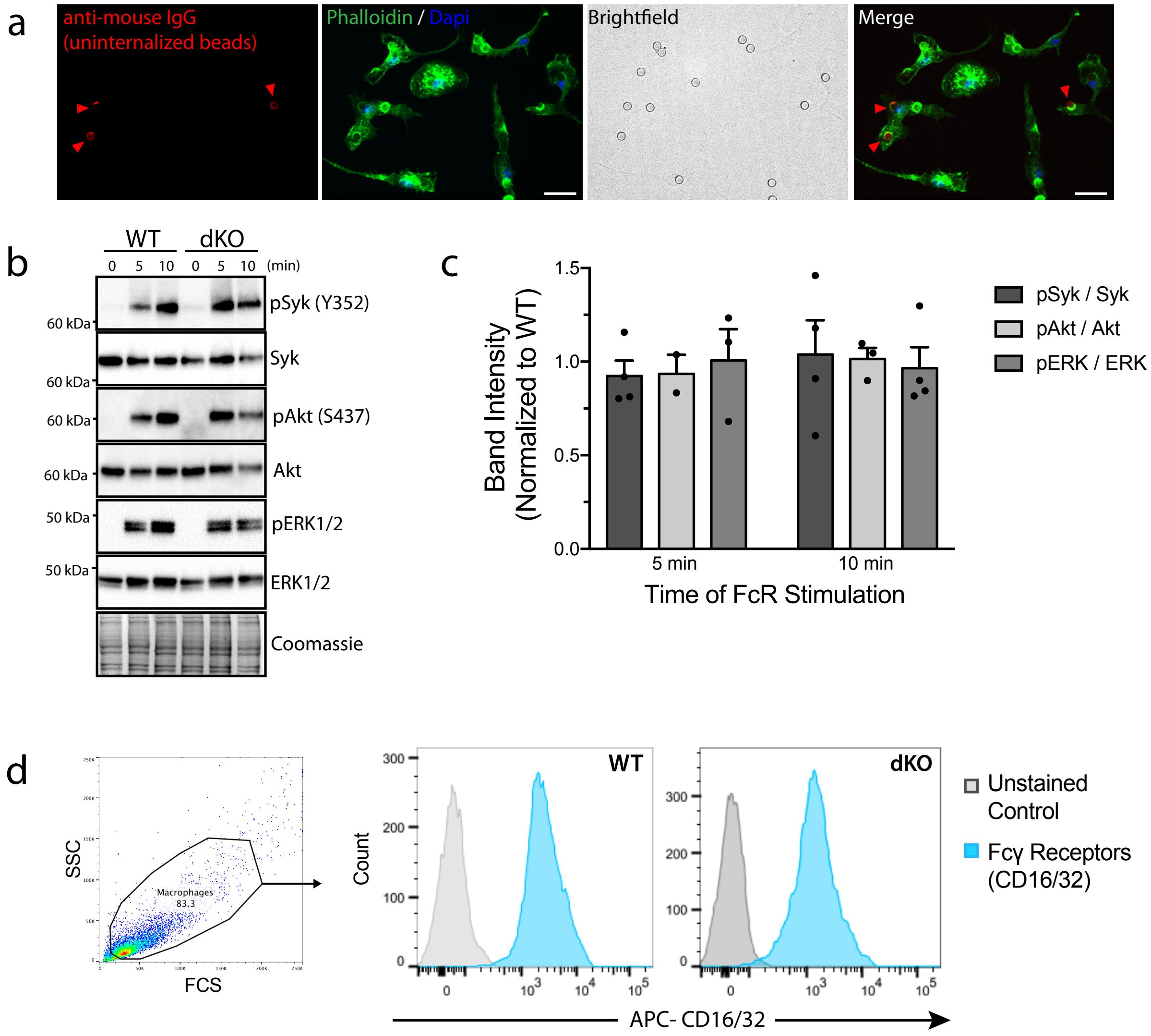

Supplementary Fig. 3: Macrophages lacking myo1e and myo1f maintain initial phagocytic signaling and normal FcR surface expression.

a) Representative image series of phagocytosis assay to quantify internalization. Cells were stained without permeabilization using anti-mouse secondary antibody to identify un-internalized beads (red arrows). This channel along with the bright field image was used to identify completely internalized beads. Cells were identified/counted using phalloidin/DAPI. Scale bar, 25 μm.

b) Representative Western blot analysis of phagocytic signaling pathways induced by FcR crosslinking in WT and dKO macrophages. The 0 timepoint represents an un-induced state.

c) Quantification (mean ± SEM) of Western blots used to examine FcR signaling, with band intensities for dKO macrophages normalized to WT values. Data from from 3-4 independent experiments, with 2-4 experiments used for quantification.

d) Flow cytometry analysis of FcRs present on the cell surface in WT and dKO macrophages.

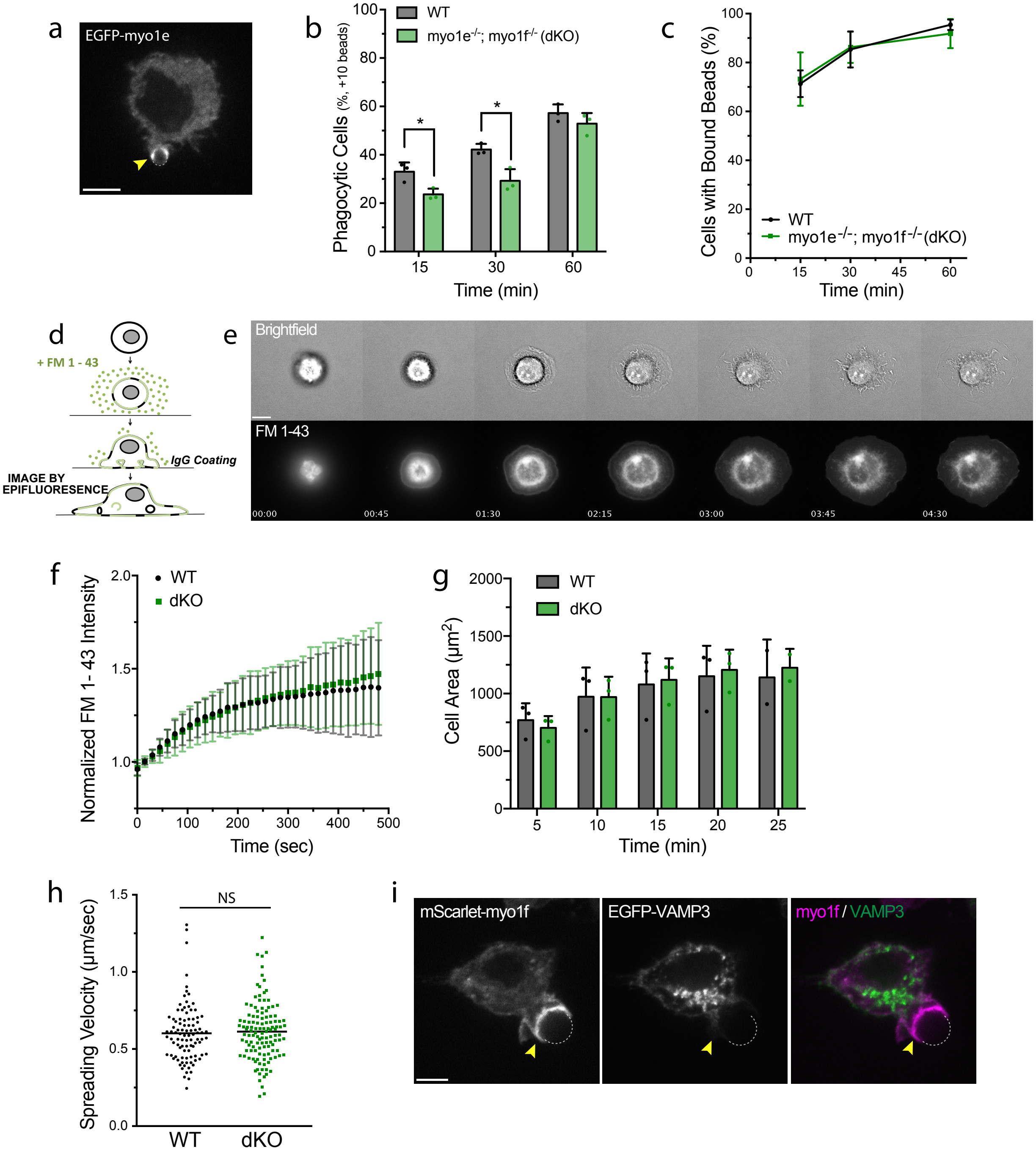

Supplementary Fig. 4: Myo1e and myo1f are not required for focal exocytosis.
a) Myo1e is recruited to phagocytic cups formed around small targets. EGFP-myo1e-transfected RAW macrophages were presented with 2 µm IgG-coated beads. Yellow arrowhead points to the phagocytic cup, and a dotted white line outlines the bead. Scale bar, 5 µm.
b) Graph of percentage (mean ± SD) of phagocytic cells, defined as those that internalized at least 10 beads. WT and dKO BMDM were challenged with 2 µm IgG-

coated beads and analyzed at 15 ($p=0.022$), 30 ($p=0.013$), 60 ($p=0.25$) minutes. Data from 3 independent experiments (*=$p<0.05$).

c) Percentage of cells (mean ± SEM) that bound at least one small bead during the phagocytosis time course of (b).

d) Schematic of the FM 1-43-based membrane quantification experiment. WT and dKO macrophages are serum-starved in suspension and briefly exposed to lipid dye FM 1-43 before being added to a flow chamber to perform frustrated phagocytosis in the presence of excess dye. Fluorescence intensity values are normalized individually to the pre-spread cell.

e) Representative time-lapse of WT BMDM performing frustrated phagocytosis and taking up FM 1-43 dye, with brightfield and epifluorescence images shown. Time stamp at lower left in minutes:seconds. Scale bar, 10 μm. See Supplementary Movie 4.

f) Graph (mean ± SD) of normalized FM 1-43 intensity during frustrated phagocytosis. Data pooled from 3 independent experiments (n = 67 WT cells, 77 dKO cells).

g) Graph of cell area (mean ± SD) during frustrated phagocytosis. WT and dKO macrophages were serum starved in suspension, plated on IgG-coated coverslips, and fixed and stained with phalloidin. Data from 3 independent experiments (>100 cells per time point).

h) Leading edge velocity of WT and dKO cells during frustrated phagocytosis was measured by kymography. Data pooled from 6 independent experiments (n = 95 WT, n = 123 dKO; $p=0.66$).

i) Myo1f and VAMP3 do not colocalize at the phagocytic cup. RAW macrophages co-transfected with EGFP-VAMP3 and mScarlet-myo1f were challenged to engulf 6 μm IgG-coated beads. Yellow arrowhead points to the phagocytic cup, and a dotted white line outlines the bead. Scale bar, 5 μm.

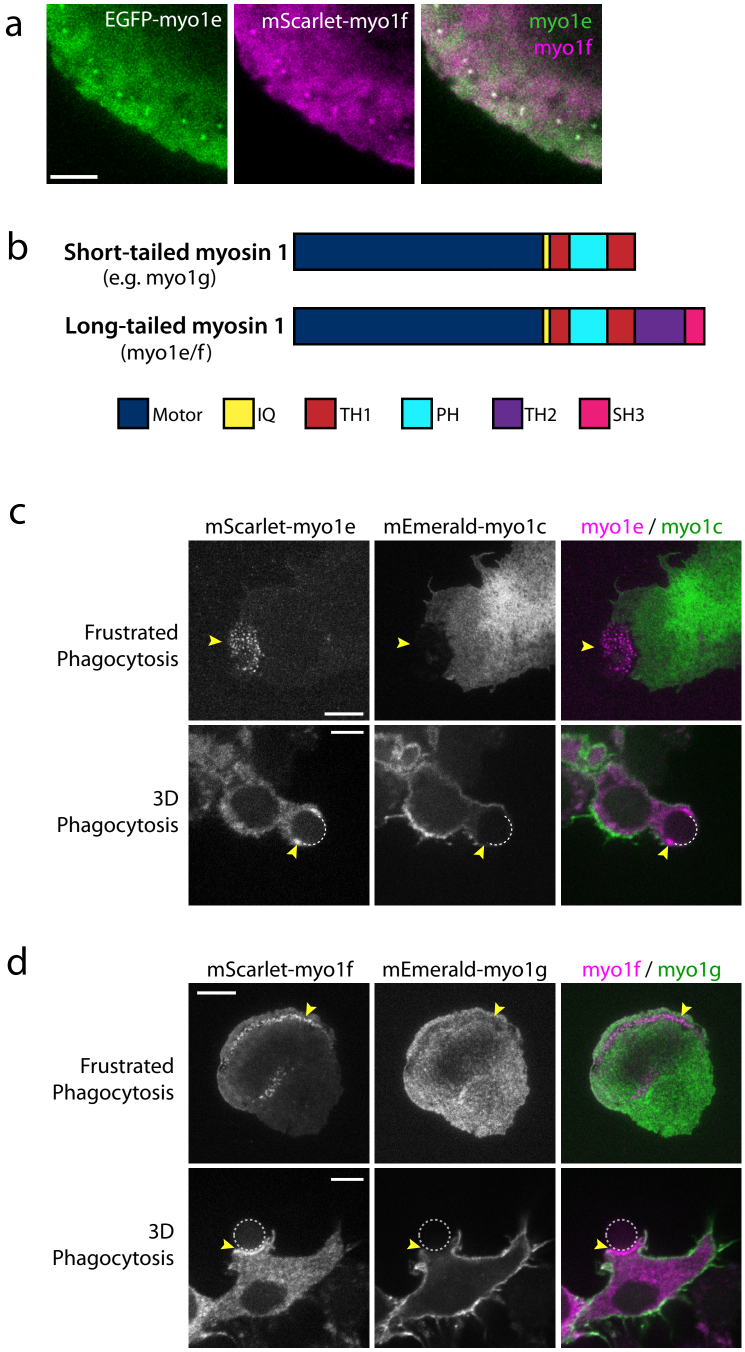

Supplementary Fig. 5: Only long-tailed class 1 myosins (myo1e/f) localize to actin-based adhesions during frustrated phagocytosis.

a) Myo1e and myo1f colocalize at punctate adhesions. Spreading edge of RAW macrophage expressing EGFP-myo1e and mScarlet-myo1f conducting frustrated phagocytosis and imaged by TIRFM. Scale bar, 5 µm.

b) Schematic depicting domain organization of short-tailed (e.g. myo1g) vs. long-tailed class 1 myosins (myo1e/f). Tail homology 1 (TH1) domain contains a putative Pleckstrin Homology (PH) domain to mediate binding to lipids. Long-tailed myosins also contain Tail Homology 2 (TH2) and Src Homology 3 (SH3) domains.

c) Myo1c does not colocalize with myo1e at FcR-actin adhesions or at the phagocytic cup. Upper panel: Representative TIRFM image of RAW macrophage co-transfected with mScarlet-myo1e and mEmerald-myo1c conducting frustrated phagocytosis. Yellow arrowhead points to the actin wave marked by myo1e. Scale bar, 10 µm. Lower panel: Representative confocal image of RAW macrophage co-transfected with mScarlet-myo1e and mEmerald-myo1c engulfing a 6 µm IgG-coated bead. Yellow arrowhead points to the phagocytic cup, and the bead is outlined by a dotted white line. Scale bar, 5 µm.

d) Myo1g does not colocalize with myo1f at FcR-actin adhesions or at the phagocytic cup. Upper panel: Representative TIRFM image of RAW macrophage co-transfected with mScarlet-myo1f and mEmerald-myo1g conducting frustrated phagocytosis. Yellow arrowhead points to the actin wave marked by myo1f. Scale bar, 10 µm. Lower panel: Representative confocal image of RAW macrophage co-transfected with mScarlet-myo1f and mEmerald-myo1g engulfing a 6 µm IgG-coated bead. Yellow arrowhead points to the phagocytic cup, and the bead is outlined by a dotted white line. Scale bar, 5 µm.

a

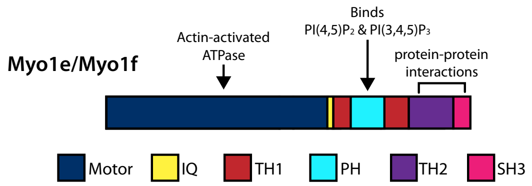

b

Myosin Construct	Description	Localizes to Actin Wave	Additional Notes
EGFP-myo1e N164A	Rigor mutant	+	
EGFP-myo1e T119I	Non-actin binding	–	Cytosolic localization
EGFP-myo1e tail	(TH1/TH2/SH3)	+	Enhanced membrane localization
EGFP-myo1e Δ TH1		+	More cytosolic than full-length
EGFP-myo1e Δ TH2		–	Cytosolic localization
EGFP-myo1e Δ SH3		+	Actin waves unstable

c

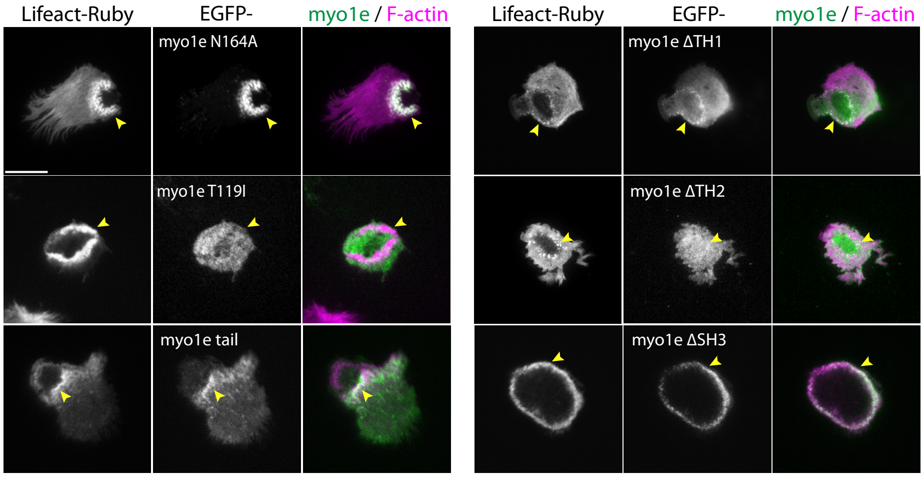

d

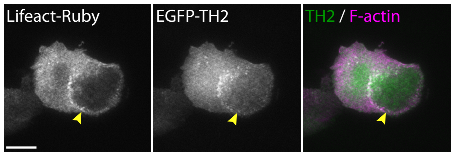

Supplementary Fig. 6: Actin-binding motor and TH2 domain of myo1e enable localization to actin-based adhesions during frustrated phagocytosis.
a) Schematic of myo1e/f protein domains. N-terminal motor domain produces force on F-actin by ATP hydrolysis. Neck region is defined by a single IQ-motif. Tail Homology 1 (TH1) domain contains a putative Pleckstrin Homology (PH) domain to mediate binding to lipids. Tail Homology 2 (TH2) and Src Homology 3 (SH3) domains make myo1e/f unique among class 1 myosins and mediate protein-protein interactions.
b) Table summarizing myosin colocalization with actin waves for myo1e mutants/deletional constructs during frustrated phagocytosis. Myo1e N164A is a rigor mutant (strongly bound to actin) and T119I is a putative non-actin-binding motor mutant.
c) Representative TIRFM images of RAW macrophages, co-expressing EGFP-tagged myo1e mutant/deletional constructs and Lifeact-Ruby, performing frustrated phagocytosis. Yellow arrowheads point to actin waves. Scale bar, 10 μm.

d) Myo1e TH2 domain is sufficient for localization to the actin wave. RAW macrophages, co-expressing EGFP-TH2 and Lifeact-Ruby, performing frustrated phagocytosis and imaged by TIRFM. Yellow arrowhead points to actin waves. Scale bar, 10 μm.

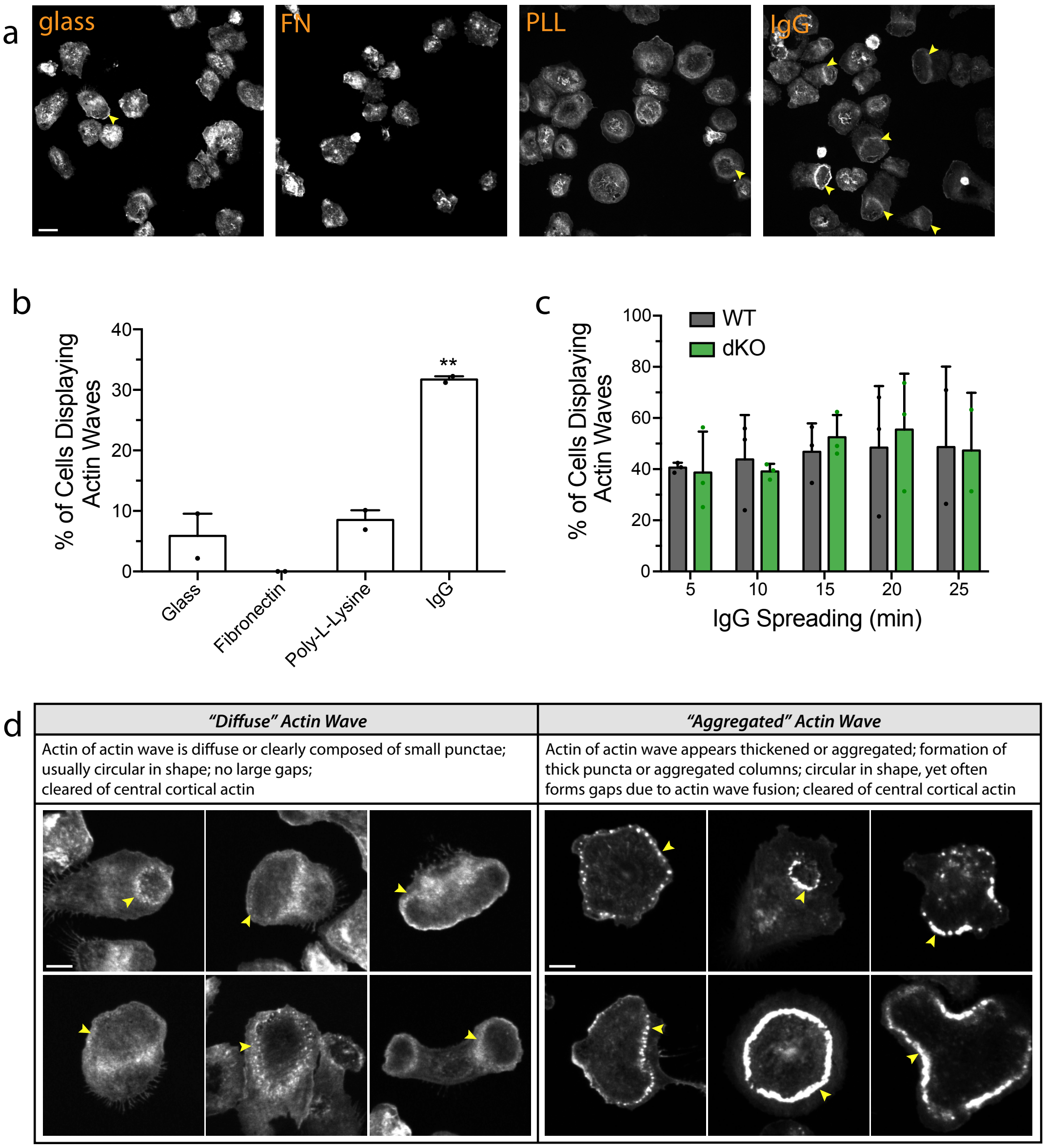

Supplementary Fig. 7: Frustrated phagocytosis in primary BMDM.
a) Representative confocal images of WT BMDM forming actin waves on differing substrates: glass, fibronectin (FN), poly-L-lysine (PLL) and IgG. Coating was performed at the following concentrations: FN (20 µg/mL), PLL (100 µg/mL), IgG (50 µg/mL). Cells were allowed to spread for 15 minutes, then fixed and stained with fluorescently-labeled phalloidin. Yellow arrowheads point to actin waves. Scale bar, 20 µm.
b) Percentage (mean ± SEM) of WT BMDM that form actin waves on different substrates. Data from 2 independent experiments. N = >200 cells judged per substrate per experiment. Percentage of BMDM that form actin waves on IgG is significantly greater than on other substrates (Glass: p=0.0029, Fibronectin: p=0.0013, Poly-L-Lysine: p=0.0044, one-way ANOVA with multiple comparisons).
c) Percentage (mean ± SEM) of cells that form actin waves is not different between WT and dKO macrophages over time. WT and dKO BMDM spread on IgG-coated

coverslips for specific time points, were then fixed and stained with fluorescently-labeled phalloidin. Data from 2-3 independent experiments (>100 cells per genotype).
d) Picture guide used to assess if actin waves of primary BMDM were “diffuse” or “aggregated”. Representative cells are BMDM conducting frustrated phagocytosis and strained with fluorescently-labeled phalloidin. Yellow arrowheads point to actin waves. Scale bar, 10 µm.

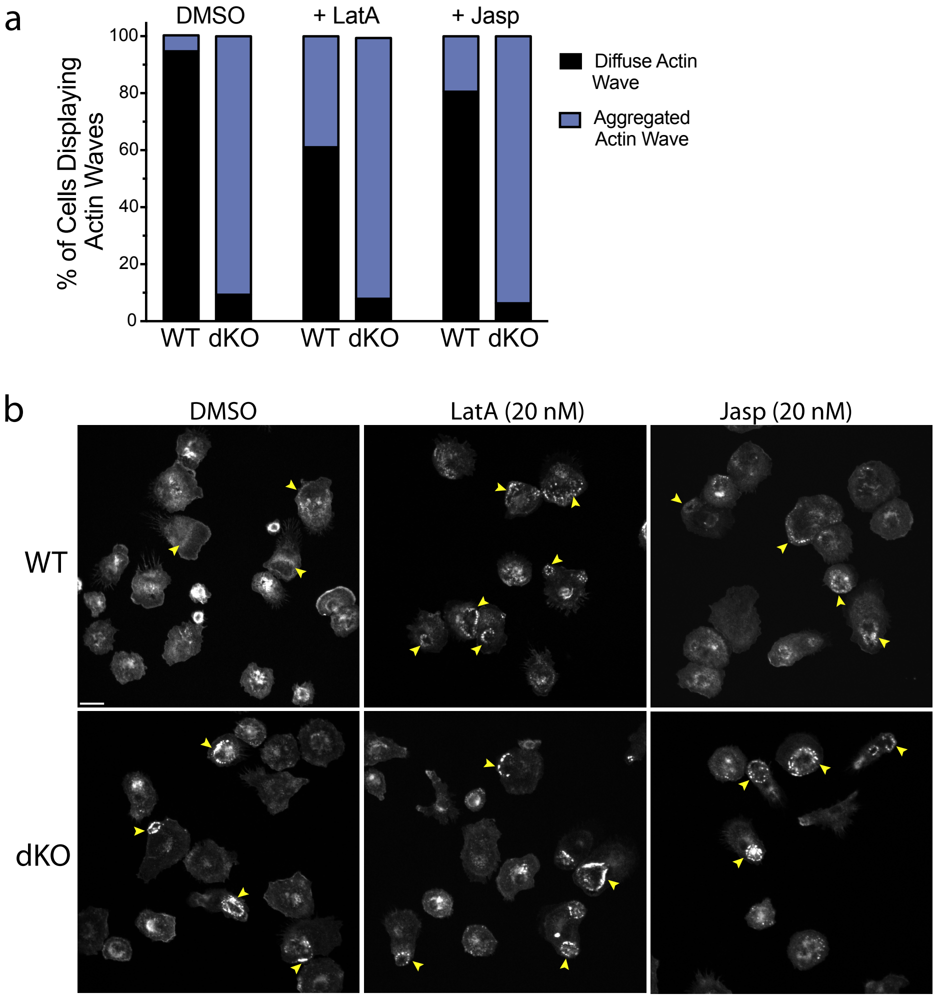

Supplementary Fig. 8: Actin disrupting drugs cannot phenocopy or rescue the defects in actin waves of dKO macrophages.

a) Treating cells with low doses of Latrunculin A or Jasplakinolide does not rescue actin wave morphology. Graph depicting the mean percentage of cells forming diffuse or aggregated actin waves in WT and dKO BMDM in the presence of 20 nM Latrunculin A or 20 nM Jasplakinolide. Cells were fixed after 10 minutes of frustrated phagocytosis and stained with fluorescently-labeled phalloidin. Data from 2 independent experiments (>70 cells per genotype, judged blindly).

b) Representative confocal images of actin waves in WT and dKO BMDM treated with F-actin drugs. Yellow arrows point to actin waves. Scale bar, 20 µm.

a

b

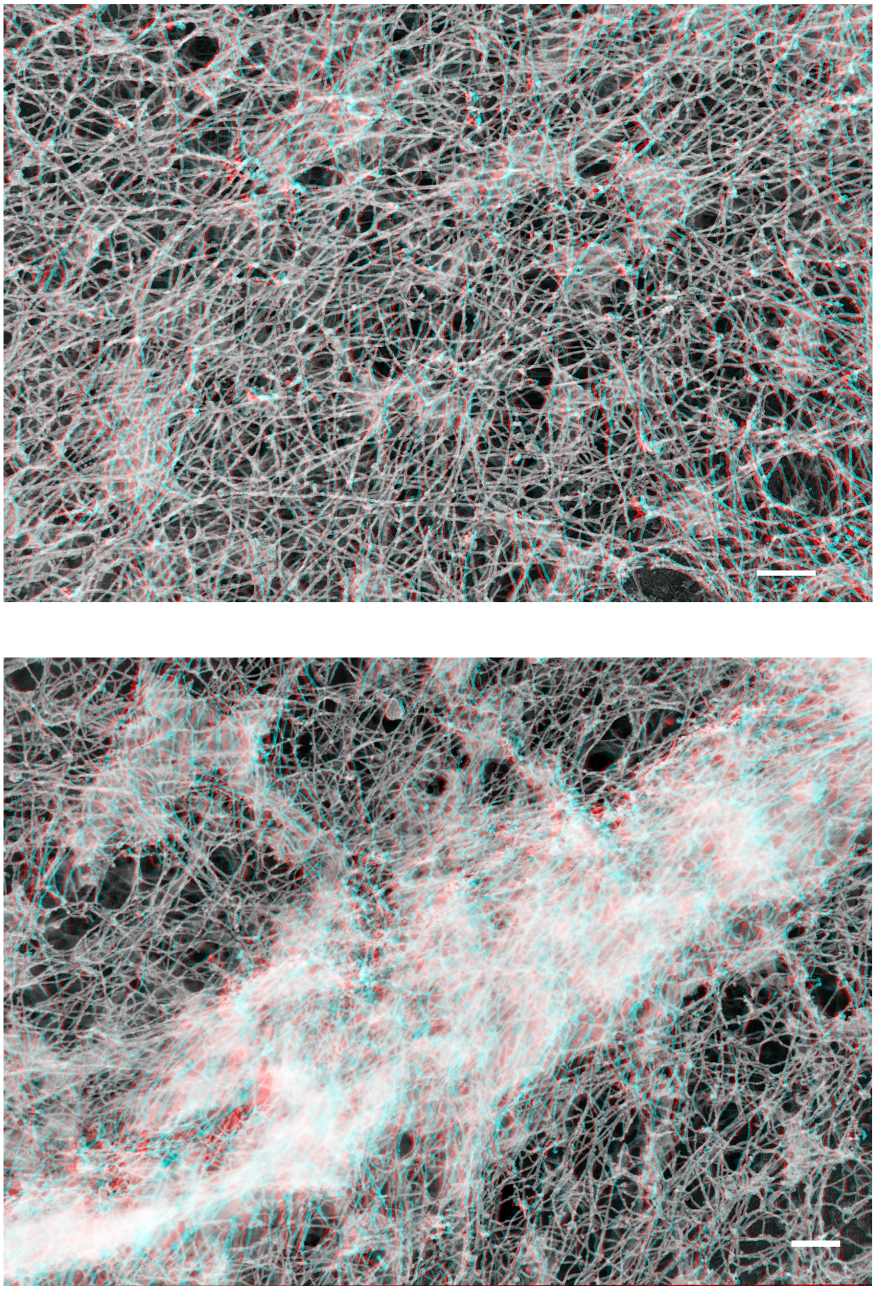

Supplementary Fig. 9: 3D platinum replica EM image of actin waves in WT and dKO macrophages.

Three-dimensional structure of representative actin wave in WT (a, corresponds to Fig. 5f) and dKO cells (b, corresponds to Fig. 5k). Overlapped stereo pairs of platinum replica electron microscopy images taken at +10 (blue) and -10 (red) degrees of sample tilt. Use 3D view glasses for volume viewing (left eye, blue). Scale bars, 0.2 µm.

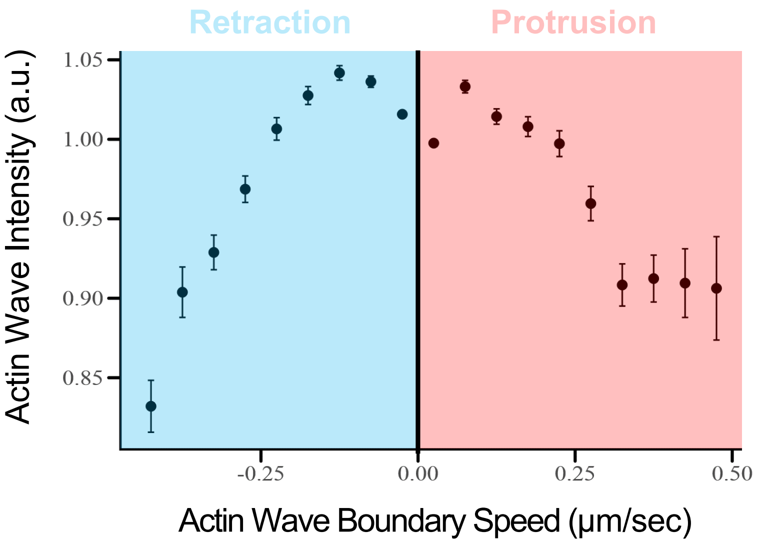

Supplementary Fig. 10: Actin adhesion tracking of dKO macrophages.
Graph (mean ± SD) showing the distribution of intensities of actin adhesions vs. actin wave boundary speed during frustrated phagocytosis in multiple dKO BMDM (n= 7 cells). dKO BMDM were transfected with EGFP-actin and imaged by TIRFM. Error bars represent variability among cells.

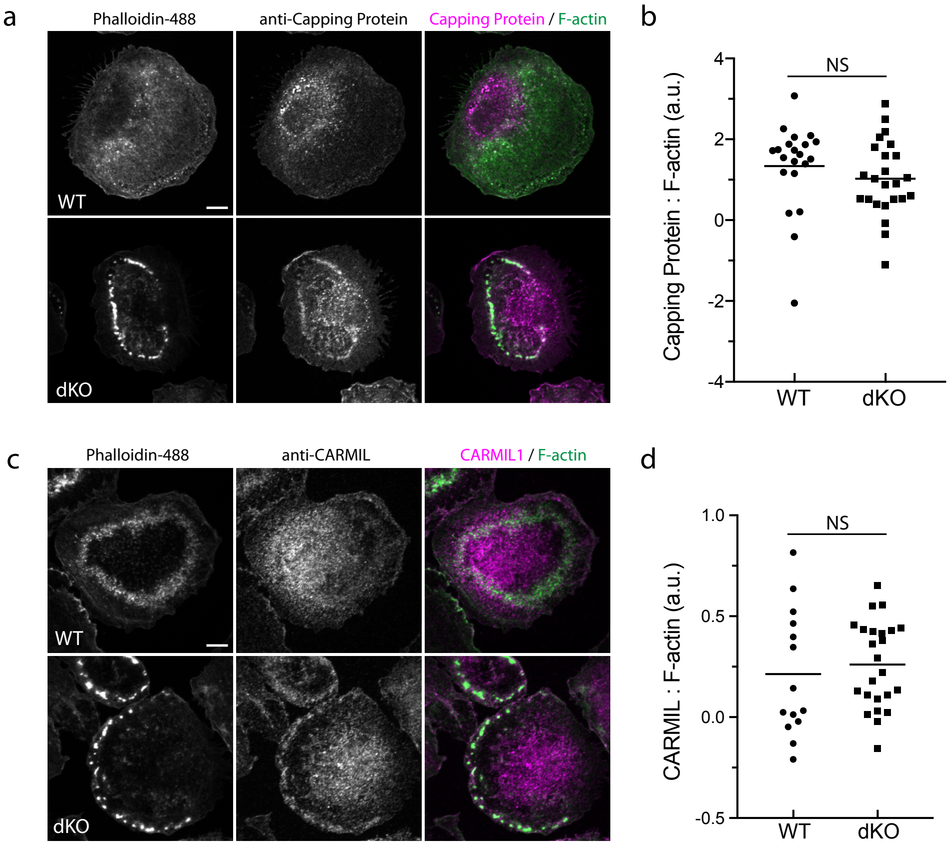

Supplementary Fig. 11: Levels of capping protein, WASP, and CARMIL appear unchanged in dKO macrophages.

a) Immunostaining of capping protein complex in WT and dKO BMDM during frustrated phagocytosis. Cells were counter-stained with fluorescently-labeled phalloidin. Scale bar, 5 µm.

d) Ratio of F-actin:capping protein in the actin wave of WT and dKO BMDM. Mean fluorescence intensity of actin wave staining was measured in ImageJ using hand drawn polygonal ROIs. Subtraction of perinuclear capping protein signal occasionally resulted in negative values, as shown on the graph. Data from 1 experiment (N = 21 WT cells, 24 dKO cells, p=0.29, unpaired t-test).

c) Immunostaining of CARMIL1 in WT and dKO BMDM during frustrated phagocytosis. Cells were counter-stained with fluorescently-labeled phalloidin. Scale bar, 5 µm.

d) Ratio of F-actin:CARMIL in the actin wave of WT and dKO BMDM. Mean fluorescence intensity of actin wave staining was measured in ImageJ using hand drawn polygonal ROIs. Subtraction of perinuclear CARMIL signal occasionally resulted in negative values, as shown on the graph. Data from 1 experiment (N = 14 WT cells, 24 dKO cells, p=0.58, unpaired t-test).

Supplementary Table 1. Primers to generate myosin 1 constructs used in this study.

Construct	Template	Primers
mEmerald-myo1e<br>tdTomato-myo1e	EGFP-C1-myo1e[1]	GTCCGGACTCAGATCT**ATGGGAAGCAAAGGTGTCTACCA**
		TAGATCCGGTGGATCC**TCAGATCTTGGTCACATAGT**
pEGFP-myo1f	Human myosin 1f (NM_012335.3)	CTCAAGCTTCGAATTCC**ATGGGCAGCAAGGAGCGCTTCCACTG**
		TAGATCCGGTGGATCC**TCAGATCTTCTCCACGTAGTTTCCTGGG**
mEmerald-myo1f<br>tdTomato-myo1f<br>mScarlet-myo1f	EGFP-myo1f	GTCCGGACTCAGATCT**ATGGGCAGCAAGGAGCGC**
		TAGATCCGGTGGATCC**TCAGATCTTCTCCACGTAGTTTCCT**
mEmerald-myo1c	YFP-C1-myo1c[*]	GTCCGGACTCAGATCT**ATGGAGAGTGCGCTCACCGCCCG**
		TAGATCCGGTGGATCC**TCACCGAGAATTCAGCCGTGGGGC**
mEmerald-myo1g	EGFP-C1-myo1g[*]	GTCCGGACTCAGATCT**ATGGAGGACGAGGAAGGCCCTGA**
		TAGATCCGGTGGATCC**TCAGCGGCTGGGCCAGAGCA**

[*]Kind gifts from Matt Tyska

References

[1]Krendel, M., Osterweil, E.K. & Mooseker, M.S. Myosin 1E interacts with synaptojanin-1 and dynamin and is involved in endocytosis. FEBS Lett 581, 644-650 (2007).
