# Supplementary material for: Membrane-cytoskeletal crosstalk mediated by myosin-I regulates adhesion turnover during phagocytosis
Source: Nat Commun. 2019 Mar 19;10:1249. doi: 10.1038/s41467-019-09104-1 (PMC6425032; doi:10.1038/s41467-019-09104-1)
Supplement: Supplementary file 3 — Description of Additional Supplementary Files [file 41467_2019_9104_MOESM3_ESM.pdf]

Supplementary Movies

Supplementary Movie 1: Myo1e leads actin polymerization during phagocytic cup closure.
Localization of myo1e (magenta) and F-actin (green) during phagocytic cup closure. RAW macrophage expressing mScarlet-myo1e and Lifeact-EGFP engulfing an 8 μm IgG-coated bead. Elapsed time is shown at bottom left in minutes:seconds. Scale bar, 5 μm.

Supplementary Movie 2. Myo1f leads actin polymerization during phagocytic cup closure.
Localization of myo1f (magenta) and F-actin (green) during phagocytic cup closure. RAW macrophage expressing mScarlet-myo1f and Lifeact-EGFP engulfing an 8μm IgG-coated bead. Elapsed time is shown at bottom left in minutes:seconds. Scale bar, 5 μm.

Supplementary Movie 3: BMDM produce tangential forces on the substrate during frustrated phagocytosis.
Representative example of traction force microscopy (TFM) experiment using primary macrophage performing frustrated phagocytosis. DIC imaging (left) alongside traction force map (right). The magnitude of the brightness in the traction map corresponds to the magnitude of the stress (i.e. a pixel value of 50 = 50 Pa), with the intensities displayed using a pseudocolor lookup table as indicated in the color map in Fig. 2a (dark blue = 0 Pa, bright red = 200 Pa). Time stamp (min) at upper right corner of DIC panel. Scale bar, 10 μm.

Supplementary Movie 4. Tracking membrane dynamics in BMDM during frustrated phagocytosis.
WT BMDM performing frustrated phagocytosis and taking up FM1-43 dye. Bright field imaging and FITC widefield imaging. Elapsed time is shown at bottom left in minutes:seconds. Scale bar, 10 μm.

Supplementary Movie 5: Myo1f localizes to leading edge puncta during frustrated phagocytosis.
Time-lapse of spreading RAW macrophage transfected with mScarlet-myo1f (inverted lookup table) conducting frustrated phagocytosis and imaged by DIC (top) and TIRF microscopy (bottom). Elapsed time is shown at bottom right in minutes:seconds. Scale bar, 10 μm.

Supplementary Movie 6-7: Myo1f colocalizes with Fcγ receptors during frustrated phagocytosis.
Time-lapse of spreading RAW macrophage transfected with EGFP-FcγRIIA (green) and mScarlet-myo1f (magenta) conducting frustrated phagocytosis and imaged by TIRF microscopy. Elapsed time is shown in minutes:seconds. Scale bar, 10 μm.

Supplementary Movie 8: The actin wave of frustrated phagocytosis is an adhesive structure.
Two RAW macrophages transfected with Lifeact-EGFP (green) were allowed to spread on IgG. By TIRFM (lower panel), one cell (upper left) is forming an actin wave, while the other is not. Cell adhesion was tested using a micropipette. The cell without an actin wave (lower right) is easily pushed around, while the cell with the wave resists detachment and appears firmly attached to substrate, as shown in the DIC microscopy image (upper panel). Only the force strong enough to rip the cell off completely results in detachment. Scale bar, 5 µm.

Supplementary Movie 9: Myo1e localizes at the tips of actin plumes within the phagocytic cup.
Revolving images of 3D reconstructions of confocal Z-stacks obtained using RAW macrophage transfected with EGFP-myo1e (green), fixed and counter-stained with phalloidin to label F-actin (magenta). Bead image is not included.

Supplementary Movie 10: Myo1f localizes at the tips of actin plumes within the phagocytic cup.
Revolving images of 3D reconstructions of confocal Z-stacks obtained using RAW macrophage transfected with EGFP-myo1f (green), fixed and counter-stained with phalloidin to label F-actin (magenta). Bead image is not included.

Supplementary Movie 11: Individual actin-based adhesions move around target during FcR-mediated phagocytosis.
Maximum intensity projection of RAW macrophage transfected with mEmerald-Lifeact (magenta) internalizing a 7 µm IgG-coated latex bead (green), imaged by lattice light sheet microscopy. Inverted F-actin channel on the right shows actin plumes dynamically progressing along the bead slightly behind the tip of the phagocytic cup. Elapsed time is shown in minutes:seconds. Scale bar, 2 µm.

Supplementary Movie 12: Three-dimensional structure of the actin waves in WT and dKO macrophages.
PREM of WT (left) and dKO (right) macrophages. The movie frames represent platinum replica electron microscopy images taken at sequential sample tilt angle from +10 to -10 degrees, step size 5 degrees. Scale bar, 0.5 µm.

Supplementary Movie 13: Dynamics of actin wave puncta in WT and dKO macrophages.
Dynamics of actin wave in WT (left) and dKO (right) macrophages. BMDM were transfected with EGFP-actin (displayed using inverted LUT) and imaged by TIRFM while performing frustrated phagocytosis. Elapsed time is shown at bottom right in minutes:seconds. Scale bar, 5 µm.

Supplementary Movie 14: Actin wave tracking analysis in dKO macrophage.
dKO macrophage transfected with EGFP-actin performing frustrated phagocytosis. Actin wave boundary speed is tracked at wave border with green color denoting

protrusion (positive changes) and yellow color denoting retraction (negative changes). The inner blue circle marks the inner boundary used for actin wave fluorescence intensity measurements. Elapsed time is shown at the bottom right in minutes:seconds. Scale bar, 10 μm.

Supplementary Movie 15: Membrane lifting observed at sites of phagocytic adhesions by TIRFM in WT macrophages
WT macrophage transfected with EGFP-actin (green) and stained with FM 4-64 (pink) performing frustrated phagocytosis and imaged by DIC and TIRF microscopy. Areas around actin-based phagocytic adhesions show reduced plasma membrane fluorescence by TIRFM. Elapsed time is shown at bottom left in minutes:seconds. Scale bar, 10 μm.

Supplementary Movie 16: Membrane lifting not observed at sites of phagocytic adhesions by TIRFM in dKO macrophages.
dKO macrophage transfected with EGFP-actin (green) and stained with FM 4-64 (pink) performing frustrated phagocytosis and imaged by DIC and TIRF microscopy. Aggregated actin adhesions are not surrounded by reduced membrane fluorescence. Elapsed time is shown at bottom left in minutes:seconds. Scale bar, 10 μm.
