# Supplementary material for: Membrane-cytoskeletal crosstalk mediated by myosin-I regulates adhesion turnover during phagocytosis
Source: Nat Commun. 2019 Mar 19;10:1249. doi: 10.1038/s41467-019-09104-1 (PMC6425032; doi:10.1038/s41467-019-09104-1)
Supplement: Supplementary file 20 — Reporting Summary [file 41467_2019_9104_MOESM20_ESM.pdf]

nature research

Corresponding author(s): Krendel, Mira

Reporting Summary

Nature Research wishes to improve the reproducibility of the work that we publish. This form provides structure for consistency and transparency in reporting. For further information on Nature Research policies, see Authors & Referees and the Editorial Policy Checklist.

Statistical parameters

When statistical analyses are reported, confirm that the following items are present in the relevant location (e.g. figure legend, table legend, main text, or Methods section).

n/a	Confirmed	
☐	☒	The exact sample size (n) for each experimental group/condition, given as a discrete number and unit of measurement
☐	☒	An indication of whether measurements were taken from distinct samples or whether the same sample was measured repeatedly
☐	☒	The statistical test(s) used AND whether they are one- or two-sided<br>*Only common tests should be described solely by name; describe more complex techniques in the Methods section.*
☒	☐	A description of all covariates tested
☒	☐	A description of any assumptions or corrections, such as tests of normality and adjustment for multiple comparisons
☐	☒	A full description of the statistics including central tendency (e.g. means) or other basic estimates (e.g. regression coefficient) AND variation (e.g. standard deviation) or associated estimates of uncertainty (e.g. confidence intervals)
☐	☒	For null hypothesis testing, the test statistic (e.g. F, t, r) with confidence intervals, effect sizes, degrees of freedom and P value noted<br>*Give P values as exact values whenever suitable.*
☒	☐	For Bayesian analysis, information on the choice of priors and Markov chain Monte Carlo settings
☒	☐	For hierarchical and complex designs, identification of the appropriate level for tests and full reporting of outcomes
☒	☐	Estimates of effect sizes (e.g. Cohen's d, Pearson's r), indicating how they were calculated
☐	☒	Clearly defined error bars<br>*State explicitly what error bars represent (e.g. SD, SE, CI)*

Our web collection on statistics for biologists may be useful.

Software and code

Policy information about availability of computer code

Data collection	LAS AF, Nikon NIS Elements, Volocity 5.4, FACSDiva Software
Data analysis	ImageJ, Fiji, FlowJo, Image Lab, Imaris 9.0, Prism Graphpad 7.0, OpenPIV

For manuscripts utilizing custom algorithms or software that are central to the research but not yet described in published literature, software must be made available to editors/reviewers upon request. We strongly encourage code deposition in a community repository (e.g. GitHub). See the Nature Research guidelines for submitting code & software for further information.

Data

Policy information about availability of data

All manuscripts must include a data availability statement. This statement should provide the following information, where applicable:
- Accession codes, unique identifiers, or web links for publicly available datasets
- A list of figures that have associated raw data
- A description of any restrictions on data availability

The manuscript does not include the types of data subject to mandatory deposition into publicly available databases. Source data are available from the authors upon request.

Field-specific reporting

Please select the best fit for your research. If you are not sure, read the appropriate sections before making your selection.

☒ Life sciences ☐ Behavioural & social sciences ☐ Ecological, evolutionary & environmental sciences

For a reference copy of the document with all sections, see nature.com/authors/policies/ReportingSummary-flat.pdf

Life sciences study design

All studies must disclose on these points even when the disclosure is negative.

Sample size	No statistical method was used to predetermine sample size. The samples sizes were based on previous studies with similar methodologies.
Data exclusions	For Figure 2, outliers were identified based on the interquartile range and removed.
Replication	All attempts at replication were successful. "independent" experiments in the use of bone marrow cells were defined as different vials of bone marrow from different animals. In the case of experiments using a cell line, "independent" means the experiment was done on a separate day with a different cell passage number.
Randomization	N/A
Blinding	During data analysis, investigators were blinded by shuffling image file names using an ImageJ plugin.

Reporting for specific materials, systems and methods

Materials & experimental systems

n/a	Involved in the study
☐	☒ Unique biological materials
☐	☒ Antibodies
☐	☒ Eukaryotic cell lines
☒	☐ Palaeontology
☐	☒ Animals and other organisms
☒	☐ Human research participants

Methods

n/a	Involved in the study
☒	☐ ChIP-seq
☐	☒ Flow cytometry
☒	☐ MRI-based neuroimaging

Unique biological materials

Policy information about availability of materials

Obtaining unique materials	All plasmids unique to this study are available upon request.

Antibodies

Antibodies used	Rabbit anti-myo1e has been previously described (Skowron et al., 1998); myo1f (Santa Cruz, B-5, #376534); Arp3 (Millipore, clone 13C9, #MABT95); BSA (Sigma, clone 3H6, #SAB5300158); rat anti-mouse CD16/32 (BD, clone 2.4G2, #553141); AffiniPure mouse anti-rat (Jackson Labs, 212-005-082); pSyk (Cell Signaling Technologies, #2701); Syk (Cell Signaling Technologies, #13198); pAkt (Cell Signaling Technologies, #4060); Akt (Cell Signaling Technologies, #4691); pERK (Cell Signaling Technologies, #4370); ERK (Cell Signaling Technologies, #9102); fluorescent secondary antibodies against mouse or rabbit (Life Technologies, #A-11001, #A-11008).
Validation	Myo1e and myo1f antibody validations were performed on cells derived from WT, single KO and double KO mice. All commercial antibody validations are available on manufacturers' websites.

Eukaryotic cell lines

Policy information about cell lines

Cell line source(s)	RAW264.7; ATCC
Authentication	No authentication procedures were used.
Mycoplasma contamination	Cell line tested negative for mycoplasma contamination.
Commonly misidentified lines (See ICLAC register)	This cell line does not appear on the list of commonly misidentified lines.

Animals and other organisms

Policy information about studies involving animals; ARRIVE guidelines recommended for reporting animal research

Laboratory animals	Myo1e-/- mice, myo1f-/- mice, and myo1e-/-; myo1f-/- double knockout (dKO) mice were maintained on a C57BL/6 background. Both male and female mice were used; for each individual experiment utilizing BMDM, bone marrow preparations from age- and sex-matched mice were used.
Wild animals	N/A
Field-collected samples	N/A

Flow Cytometry

Plots

Confirm that:

☒ The axis labels state the marker and fluorochrome used (e.g. CD4-FITC).

☒ The axis scales are clearly visible. Include numbers along axes only for bottom left plot of group (a 'group' is an analysis of identical markers).

☒ All plots are contour plots with outliers or pseudocolor plots.

☒ A numerical value for number of cells or percentage (with statistics) is provided.

Methodology

Sample preparation	Cultured BMDM were pelleted (250 x g, 5 min, 4°C, 1 x 10^6 cells/tube) and resuspended in fresh FACS buffer (5% FBS/PBS/0.1% NaN3) and blocked with rat anti-mouse CD16/32 (Biolegend, clone 93, #101301) for 30 minutes on ice. Cells were then incubated with the specified fluorophore-conjugated antibodies for 50 minutes in the dark. Cells were washed twice and resuspended in 0.2 mL FACS buffer for immediate analysis.
Instrument	BD LSR II
Software	BD FACSDiva Software was used for data collection. Data was processed using FlowJo Software.
Cell population abundance	N/A
Gating strategy	Given that the BMDM culturing protocol produces a near homogeneous macrophage population, gating was performed once using light scatter properties to avoid cellular debris and clumped/irregular cells.

☒ Tick this box to confirm that a figure exemplifying the gating strategy is provided in the Supplementary Information.
